# Improving access to multi-drug resistant tuberculosis diagnostic and health services for refugees and migrants

**DOI:** 10.1186/s12916-018-1218-0

**Published:** 2018-11-30

**Authors:** Alimuddin Zumla, Ibrahim Abubakar

**Affiliations:** 1 Division of Infection and Immunity, Centre for Clinical Microbiology, University College London, London, UK; 2 NIHR Biomedical Research Centre, University College London Hospitals NHS Foundation Trust, London, UK; 3 UCL Institute for Global Health, University College London, London, UK

**Keywords:** Tuberculosis, multi-drug resistant, refugees, migrants

## Abstract

By the end of 2017, an estimated 68.5 million people were displaced from their homes worldwide, of whom 25.4 million were refugees. The transmission and globalization of multi-drug resistant tuberculosis during refugee migration is a now priority issue in the public health agenda. Political and scientific commitment at the highest national and international levels will be critical to intensifying action in promoting improved health services for migrants and refugees.

## Vulnerability of refugees to tuberculosis (TB)

The 2018 United Nations High Commission for Refugees Report indicates that, by the end of 2017, an estimated 68.5 million people had been displaced from their homes worldwide, including 25.4 million external refugees and 43.1 internally displaced people escaping conflict and persecution [1]. These figures represented an overall increase of 2.9 million people compared to those of 2016. Over the past 6 years, a major exodus of men, women, and children has been reported from Syria, Myanmar, Bangladesh, Iraq, Yemen, Sudan, Burundi, Central African Republic, the Democratic Republic of the Congo, and Ukraine, among other countries [1]. Due to the adverse living conditions, close contact, poor nutrition, and mental and physical stress, refugees are at an increased risk of acquiring a range of infectious diseases, in particular diarrheal diseases, hepatitis, arthropod-borne and waterborne parasitic diseases, and respiratory tract infections, including TB [2]. The combination of these risk factors and poor access to TB health services increases refugees’ vulnerability to acquiring TB infection, which then progresses to TB disease. Additionally, delays in diagnosis results in poor treatment outcomes and continued transmission in the community. The refugee crisis, coupled with the emergent transmission and globalization of antibiotic-resistant pathogens, including multi-drug resistant TB (MDR-TB), during mass gatherings and migration, have now become a priority issue in the international public health agenda [3, 4].

## Global TB rates

The World Health Organization’s (WHO) annual Global Tuberculosis Report of 2018 indicates that TB remains the most important cause of death from an infectious disease worldwide [5]. An estimated 10 million people developed TB in 2017, with a further 4 million people with TB remaining undiagnosed and untreated. At the current rates of global TB control efforts, WHO’s goal to end the global TB epidemic by 2030 will not be achieved [6]. Further, the paucity of data on the incidence of most diseases, including TB, among migrants and refugees hampers public health action. For example, the WHO TB Report provides no specific data on global TB rates in refugees or migrant populations. Additionally, there are an estimated 1.4 billion people worldwide with latent TB infection (LTBI), in whom TB could reactivate in conditions of stress or immunosuppression. Indeed, a significant proportion of active TB cases result from reactivation of LTBI in migrants under stress, with poor nutrition and living in inadequate conditions. Therefore, the issue of ensuring optimal TB services for refugees (which should be an integral part of the health services they receive) in order to ensure the health of the wider community has been long debated [7–9].

## Global spread of multidrug-resistant (MDR) TB

In 2017, an estimated 558,000 people developed drug-resistant TB [5], of whom an estimated 458,000 had MDR-TB (defined as resistance to the two first-line TB drugs, rifampicin and isoniazid). As a result, only half of patients with MDR-TB are cured after receiving appropriate treatment. MDR-TB continues to spread globally and is a major threat to global health security. Indeed, it is estimated that, over the next 35 years, MDR-TB will result in the deaths of approximately 75 million people [10]. MDR-TB treatment is expensive, associated with toxic side-effects, and observed therapy and dedicated patient support are required to ensure adherence and cure as well as to minimize the risk of emergence of extensively drug-resistant TB [11].

## MDR-TB in refugees

Accurate data on the global prevalence of MDR-TB in refugee and displaced populations worldwide is not available. Despite being recognized as a priority global health issue, accurate global data on the epidemiology, transmission dynamics, access to health services, and impact of TB in refugees on local health services and populations in developing countries have not been readily forthcoming [12]. The past 6 years have seen waves of refugees migrating to Europe. In anticipation of the threat of importation of infectious diseases to western Europe, a flurry of activity by public health systems have established, screening procedures for refugees and migrants from eastern Europe, Africa, Asia, and the Middle East. Nevertheless, an increasing number of reports on TB in migrants and refugees within Europe shows that the hysteria surrounding the importation of MDR-TB by refugee populations appears to be unfounded [13, 14]. In a systematic evaluation of data on MDR-TB and migration in Europe [15], all MDR-TB cases diagnosed in Austria, the Netherlands, and Norway, as well as a high proportion of those reported in other European countries (90% in the UK, 89% in France, 87% in Italy, and 94% in Germany), occurred in migrants. However, these refugees were already at higher risk of contracting MDR-TB in their country of origin, where high MDR-TB rates prevail. A study from Norway showed that migrants from high TB-incidence countries had little influence on the transmission of TB in Norway [16].

In the *BMC Medicine* Migrant and Refugee Health collection (https://www.biomedcentral.com/collections/migrant-and-refugee-health), two studies discuss the issue of refugees and MDR-TB. Pescarini et al. [17] describe a cross-sectional study in a central area of Sao Paulo, Brazil, focusing on migration and TB transmission utilizing molecular genotyping. Cross-transmission between migrants and Brazilians was confirmed, yet the contribution by migrants was not substantial. Nellums et al. [18] conducted a systematic review and metanalyses of 15 selected studies reporting MDR-TB treatment outcomes for 258 migrants and 174 non-migrants in European countries. The estimated rate of adherence to MDR-TB treatment across all patients was 71% . MDR-TB treatment adherence rates among migrants in high-income, low TB-incidence countries approached global targets for treatment success (75%) and were comparable to rates in non-migrants. Therefore, approximately 70% of migrant and non-migrant patients appear to adhere to MDR-TB treatment. Nevertheless, only 50% of MDR-TB patients worldwide successfully complete treatment, with nearly 25% being lost to follow-up [5]. This indicates the importance of increasing adherence by reducing the associated social risk factors, which in turn could achieve higher MDR-TB treatment completion rates and enable reductions in transmission.

## TB screening in refugees

Urgent calls for action have been voiced to ensure access to early diagnosis and care of TB among refugees [9]. Indeed, most high-income countries now have dedicated refugee TB screening policies. A survey related to TB control programs in 29 member states of the Organization for Economic Cooperation and Development in 2010 [7] showed that 86% and 55% of these countries had TB screening programs for active TB and LTBI, respectively. Ideally, baseline TB screening should be completed for every refugee child and adolescent on arrival, with a subsequent follow-up over the next 2 years. This approach creates substantial pressure on the limited healthcare resources of ‘host countries’. Therefore, a comprehensive framework for the prevention and reduction of the TB burden among migrants and refugees, in line with the WHO End TB strategy, has been suggested [2]. The framework emphasizes the conduct of priority operational research to define the TB burden in refugee situations, the challenges of access to healthcare, and the biomedical, social and structural determinants of TB across the full migration pathway, as well as a cascade of care policies ranging from the prevention to the diagnosis and treatment of TB. The data would form the basis of a developing migrant- and refugee-sensitive care and prevention program, with supportive intersectoral policies and systems.

Political commitments at the highest national and international levels provide hope for the improvement of the currently appalling status quo of global TB [19]. Following the first global ministerial conference on TB in Moscow in 2017 [20], and in advance of the UN high-level meeting on TB in 2018, the WHO, the Stop TB Partnership, and The Global Fund to Fight AIDS, Tuberculosis and Malaria launched a joint initiative to scale-up the End TB response towards universal access to TB prevention and care. The “FIND. TREAT. ALL. #EndTB” initiative [21] aims to scale-up the global response towards universal access to TB prevention and care, with an ambitious target to diagnose, treat, and report 40 million people with TB, including 3.5 million children and 1.5 million people with drug-resistant TB, between 2018 and 2022.

## Conclusions

Achieving the WHO TB control targets will require the combined efforts of many stakeholders, including donors, governments, civil society, advocacy groups, TB-affected communities, the private sector, and global public health bodies, to accelerate access to prevention and care as well as to monitor progress [19]. The initiative encompasses all countries, with priority given to the 30 high TB burden countries listed in the WHO 2018 Global TB Report [5]. It is critical that the specific issue of TB in refugees is incorporated and implemented in these initiatives. Achieving universal coverage and access to healthcare is an acknowledged human right, as enshrined in the United Nations Universal Declaration of Human Rights Article 25. Political and scientific commitment at the highest national and international levels will be critical to intensifying action in promoting the health of migrants on the road to achieving the WHO End TB targets.
